# Mite Infestation on *Rattus tanezum* rats in southwest China concerning risk models

**DOI:** 10.3389/fvets.2025.1519188

**Published:** 2025-03-14

**Authors:** Ru-Jin Liu, Xian-Guo Guo, Pei-Ying Peng, Yan Lv, Peng-Wu Yin, Wen-Yu Song, Rong Xiang, Yan-Ling Chen, Bei Li, Dao-Chao Jin

**Affiliations:** ^1^Yunnan Provincial Key Laboratory for Zoonosis Control and Prevention, Institute of Pathogens and Vectors, Dali University, Dali, China; ^2^Institute of Microbiology, Qujing Medical College, Qujing, China; ^3^Institute of Entomology, Guizhou University, Guiyang, China

**Keywords:** chigger mite, ecology, gamasid mite, infestation, logistic regression, rodent, statistic model

## Abstract

**Objective:**

The Asian house rat (*Rattus tanezumi*) is an important infectious source and reservoir host for many zoonotic diseases, and its associated mites (chigger mites and gamasid mites) can act as vectors for these diseases. The present study aimed to elucidate the infestation patterns and related ecology of the mites on the body surface of *R. tanezumi* in southwest China and explore relevant risk models.

**Methods:**

Field surveys and taxonomic identification of the mites were conducted across five provincial regions in southwest China between 2001 and 2022. The constituent ratio (*C_r_*), prevalence (*P_m_*), mean abundance (*MA*), and mean intensity (*MI*) were calculated to reflect the mite infestation status. The species richness index (*M_f_*), Shannon–Wiener diversity index (*H´*), Pielou’s evenness (*E*), and Simpson’s dominance index (*D*) were used to analyze the mite communities. A multiple logistic regression model was employed to identify potential risk factors associated with the mite infestation. The “corrplot” R software (version 4.3.1) package was used to analyze interspecific relationships among some mite species.

**Results:**

A total of 75,023 mites were collected from 3,114 *R. tanezumi* rats, representing 12 families, 46 genera, and 252 species. Among these, 173 were the chigger mite species and 79 were the gamasid mite species. The species richness and community diversity of the chigger mites were higher than those of the gamasid mites, but the infestation indexes of the gamasid mites on the rats were higher than those of the chigger mites. Several vector mite species co-existed on *R. tanezumi*, with *Laelaps nuttalli*, *L. echidninus,* and *Leptotrombidium deliense* identified as the three dominant mite species, exhibiting high infestation indexes. The multiple logistic regression analysis showed that the mite infestation was influenced by a series of environmental factors and host-related factors (potential risk factors), with temperature and relative humidity identified as the most important risk factors. The impact of these potential risk factors on the infestation of a single mite group (chigger mites or gamasid mites) was different from the impact on the co-infestation of both mite groups together. Based on the logistic regression analysis, three predictive models were developed to predict the risk probability of each *R. tanezumi* rat being infested with chigger mites alone, gamasid mites alone, and both mite groups together. A positive correlation existed between any two of the following species: *L. deliense*, *L. rubellum,* and *L. imphalum*.

**Conclusion:**

*Rattus tanezumi* rats are highly susceptible to mite infestation, hosting a variety of mite species and multiple vector mite species. The presence of multiple vector mite species on these rats increases the potential risk of transmission and persistence of related zoonotic diseases. A series of environmental factors and host factors, especially temperature and relative humidity, can influence mite infestation. The predictive models developed can estimate the likelihood of each rat being infested with mites. Some mite species show a preference for co-existing on *R. tanezumi*.

## 1 Introduction

Rodents (rats, mice, voles, etc.) often harbor two groups of rodent-associated mites—chigger mites and gamasid mites—on their body surface (1, 2). Chigger mites belong to the suborder Trombidiformes, order Acariformes, subclass Acari, and class Arachnidia within the phylum Arthropoda, with over 3,000 species recorded globally (3, 4). Gamasid mites belong to the suborder Mesostigmata (or Gamasida) and order Parasitiformes within Acari, with more than 8,000 species known worldwide (5, 6). Rodents are not only pests in agriculture and forestry but also infectious sources of many zoonotic diseases (zoonoses) (7, 8). Rodent-associated mites can act as vectors or reservoir hosts for a number of zoonotic diseases (1, 9). Chigger mites are the exclusive vector of scrub typhus (tsutsugamushi disease), and some of them can act as potential vectors of hemorrhagic fever with renal syndrome (HFRS) (10, 11). Gamasid mites can act as vectors, potential vectors, or reservoir hosts for rickettsialpox, HFRS, and other zoonotic diseases (2, 12). Southwest China, covering five provincial regions—Sichuan, Chongqing, Guizhou, Yunnan, and Tibet (Xizang Autonomous Region)—is characterized by its vast territory, complex topography, diverse ecological environments, and different climate types. It has become a hot spot for ecological research on plants and animals (13, 14). In addition, southwest China is an important focus for scrub typhus, HFRS, and other zoonotic diseases, with serious epidemics occurring in some local areas (15, 16). The Asian house rat, *Rattus tanezumi* (Temminck, 1844), is a very common rodent species with a large population and wide distribution in southwest China. In addition to causing significant damage to agricultural and forestry plants, *R. tanezumi* also serves as an animal infectious source and reservoir host for many zoonotic diseases, including scrub typhus and HFRS (8, 17). Similar to most species of rodents, *R. tanezumi* is an important host for both chigger mites and gamasid mites. Through their biting activity, these mites can transmit and preserve pathogens of zoonotic diseases among rats (e.g., *R. tanezumi*), other rodents, and even from rodents to humans (8, 18).

Due to the medical significance of rodents and their associated mites, it is very important to study the infestation and related ecology of the mites on the body surface of rodents including *R. tanezumi*. However, the taxonomic identification of chigger mites and gamasid mites (especially chigger mites) is challenging due to their tiny size and the large number of species. To accurately identify these two groups of mites at the species level, numerous careful observations and comparisons must be conducted under a microscope. Many fine structures of chigger mites need to be measured individually under high power (×400) and oil immersion (×1,000), making the identification process particularly challenging. This challenging taxonomic identification process often makes it difficult to study both mite groups simultaneously (2, 19). In some previous studies, chigger mites and gamasid mites on rodents were separately analyzed and reported and they were not regarded as a whole mite community (8, 20). Although chigger mites and gamasid mites belong to different taxonomic groups in zoological taxonomy, they often coexist on the body surface of rodents to form a mite community (21, 22). Therefore, it is necessary to study both mite groups as a whole, including their co-infestation and the related ecological aspects of the entire mite community.

Based on field surveys and taxonomic identification conducted in southwest China between 2001 and 2022, this study is the first to examine chigger mites and gamasid mites on *R. tanezumi* rats as a whole. The present study aimed to elucidate the species composition, infestation status, related ecology, and risk models of these two mite groups on *R. tanezum* in southwest China, providing scientific information and guidance for the surveillance and control of vector mites and their rat hosts.

## 2 Materials and methods

### 2.1 Field survey and collection and identification of mites

**Figure 1:**
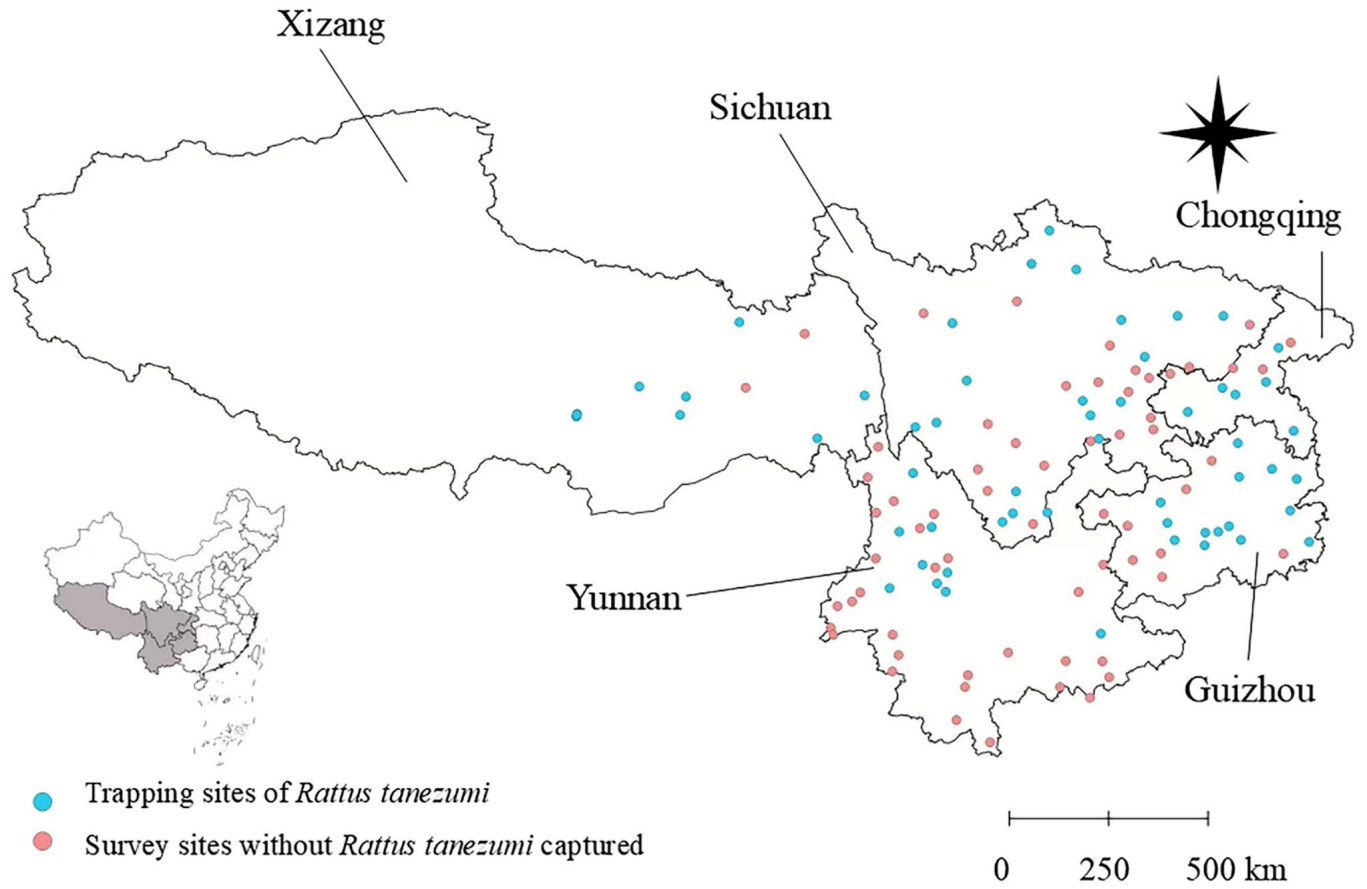
A total of 112 survey sites and 63 positive sites where the Asian house rats (*Rattus tanezumi*) were captured across the five provincial regions of southwest China between 2001 and 2022. This map was created using the standard map no. GS (2019) 1822, downloaded from the national standard map service system. The base map remained unchanged, and the geographical coordinate system was WGS84.

Between 2001 and 2022, field surveys and collections of rodent-associated mites (chigger mites and gamasid mites) were conducted at 112 survey sites across five provincial regions in southwest China (21°08′-33°41’N, 97°21′-110°11′E). The five provincial regions were Yunnan, Guizhou, Sichuan, Chongqing, and Tibet (Xizang Autonomous Region). However, the 112 survey sites did not cover the vast western part of Tibet due to its expansive and sparsely populated territory, relatively inconvenient transport, plateau hypoxia, potential risks in alpine areas, and limitations in manpower and financial support (see Figure 1 in “Results”). At each survey site, rodent hosts were routinely captured using metal wire mousetraps (18 × 12 × 9 cm, Guixi Mousetrap Apparatus Factory, Guixi, Jiangxi, China) in the afternoon or evening (20, 23). The captured rodent hosts were collected in white cloth bags the following morning and transported to a temporary field laboratory for mite collection. Each rodent host was routinely anesthetized with ether and placed on a large white square plate. Chigger mites and gamasid mites were then collected from the host’s body surface using a magnifier. The collected mites were preserved in 70% (or 75%) ethanol for subsequent use (11, 24). After each host was examined, the white square plate, along with the mousetrap, was cleaned with water and then disinfected with 75% ethanol. The white cloth bags, however, were soaked in a 5% Lysol solution for at least 24 h for disinfection and then rinsed with clean water (25–27). After the collection of the mites, each rodent host was identified to the species level according to its morphology (28–31). In the laboratory, the preserved mites were prepared as glass slide specimens using Hoyer’s medium after undergoing dehydration, transparency processing, and drying. Using taxonomic books and literature with taxonomic keys and detailed morphological descriptions, each mite specimen was eventually identified to the species level under a microscope (Olympus Corporation, Tokyo, Japan) (4, 32–37). After the identification of all the mites and their rodent hosts, *R. tanezumi* and its associated mites were selected as the subject of the present study. The use of animals (including rodent anesthesia and euthanasia) for our research was officially approved by the Animals’ Ethics Committee of Dali University, and the representative specimens were deposited in the specimen repository of the Institute of Pathogens and Vectors, Dali University.

### 2.2 Statistics of the mite infestation and community structure

Based on the taxonomic identification, the number of the species and individual mites was counted. Following conventional methods, the constituent ratio (*C_r_*), prevalence (*P_m_*), mean abundance (*MA*), and mean intensity (*MI*) were calculated to assess the mite infestation status on *R. tanezumi* (19, 38). A chi-squared test was used to assess the statistical significance of *P_m_*, while the non-parametric Kruskal–Wallis test was used to assess the statistical significance of *MA* and *MI*. A *p*-value of <0.05 was considered statistically significant; otherwise, the result was deemed non-significant. The species richness index (*M_f_*), Shannon–Wiener diversity index (*H′*), Pielou’s evenness (*E*), and Simpson’s dominance index (*D*) were calculated to analyze the structure of the mite community (2, 27).


$$C_{r}=\frac{N_{i}}{N}\times 100\%$$

$$P_{m}=\frac{H_{i}}{H}\times 100\%$$

$$MA=\frac{N_{i}}{H}$$

$$MI=\frac{N_{i}}{H_{i}}$$

$$M_{f}=\frac{S-1}{\ln N}$$

$$H^{\prime }=-\overset{S}{\underset{i=1}{\sum }}\left(\frac{N_{i}}{N}\right)\ln\left(\frac{N_{i}}{N}\right)$$

$$E=\frac{H^{\prime }}{\ln S}$$

$$D=\overset{S}{\underset{i=1}{\sum }}{\left(\frac{N_{i}}{N}\right)}^{2}$$

In the above formulas, *S is* the number of the species in the community, *N_i_* is the number of a certain species (species *i*), *N* is the total number of all the species, *H_i_* is the number of the hosts infested with mites, and *H* is the total number of the hosts examined.

### 2.3 Calculation of the host relative fatness

The relative fatness (*K*) was used to evaluate the nutritional status of the hosts. A higher relative fatness value indicates better nutritional status, while a lower value suggests poorer nutritional status. The formula for calculating the relative fatness was as follows:


$$K=\frac{100W}{L^{3}}$$

In the above formula, *K* = relative fatness (g/cm^3^), *W* = body weight (g), and *L* = body length (cm).

### 2.4 Multiple logistic regression analysis

Multiple logistic regression analysis was conducted using SPSS 23.0 to analyze potential risk factors that may influence the mite infestation on *R. tanezumi*. In the analysis, the infestation status of the mites on *R. tanezumi* was regarded as the response variable (dependent variable), with infestation defined as 1 and non-infestation as 0 (1 = infested, 0 = uninfested). The explanatory variables (independent variables) included environmental factors and host factors. The backward stepwise logistic regression method was used to remove factors that were not statistically significant, and the remaining effective factors were included in the multiple logistic regression model (39, 40). Each factor was analyzed with the first level assumed as the reference (*OR* = 1), against which the other levels were compared and *OR* values were calculated. Statistically significant factors were screened (*p* < 0.05 was considered statistically significant, otherwise not) (41, 42).


$$\mathrm{Logit}\left(P\right)=\ln\left[\frac{P}{1-P}\right]=\beta_{0}+\beta_{1}x_{1}+\beta_{2}x_{2}+\ldots +\beta_{n}x_{n}$$

In the above formula, *P* is the probability of the mite infestation and *β_0_* is a constant. The *β_1_*, *β_2_*, … and *β_n_* represent partial regression coefficients, and *x_1_*, *x_2_*, … and *x_n_* stand for the independent variables.

### 2.5 Establishment of the predictive model

Referring to the logic of the points system, a series of new partial regression coefficients (*S_n_*) were calculated. The integer parts of the newly produced partial regression coefficients (*S_1_*, *S_2_*, …, *S_n_*) were then used in the following calculation for the risk score (*S_c_*). Based on the calculated risk score (*S_c_*), risk assessment models for the probability of the mite infestation on *R. tanezumi* were finally established using the following formulas (40, 41, 43):


$$S_{n}=\frac{\beta_{n}}{\beta_{\text{min}}}$$

$$S_{c}=S_{1}+S_{2}+S_{3}+\ldots S_{n}$$

$$P^{\prime }=\frac{1}{1+\exp^{\left[-\left(\beta_{0}+S_{c}\beta_{\text{min}}\right)\right]}}$$

In the above formulas, *β_min_* represents the smallest partial regression coefficient from the multiple logistic regression analysis, *S_c_* is the sum of the risk scores (the sum of the newly produced partial regression coefficients, *S_1_*, *S_2_*, …, *S_n_*), and *P′ is* the risk probability of the mite infestation on *R. tanezumi*.

### 2.6 Analysis of the interspecific relationship among the different mite species

Based on Spearman’s correlation coefficient (*r*), the “corrplot” statistical package in R software (version 4.3.1) was used to visualize the interspecific relationships among some vector species of the mites on *R. tanezumi* (44, 45).

## 3 Results

### 3.1 Survey sites and number of *Rattus tanezumi* rats

A total of 3,114 Asian house rats (*R. tanezumi*) were captured from 63 of the 112 survey sites across the five provincial regions of southwest China, including Yunnan, Guizhou, Sichuan, Tibet (Xizang), and Chongqing (Figure 1). Among the five provincial regions, Yunnan had the largest number of the rats, accounting for 62.30% of the total (constituent ratio *C_r_* = 62.30%, 1940/3114), followed by Sichuan (*C_r_* = 22.13%, 689/3314), Guizhou (*C_r_* = 10.85%, 338/3114), and Chongqing (*C_r_* = 3.28%, 102/3114). Tibet had the lowest number of the *R. tanezumi* rats (*C_r_* = 1.44%, 45/3114).

### 3.2 Mite composition on *Rattus tanezumi*

**Figure 2:**
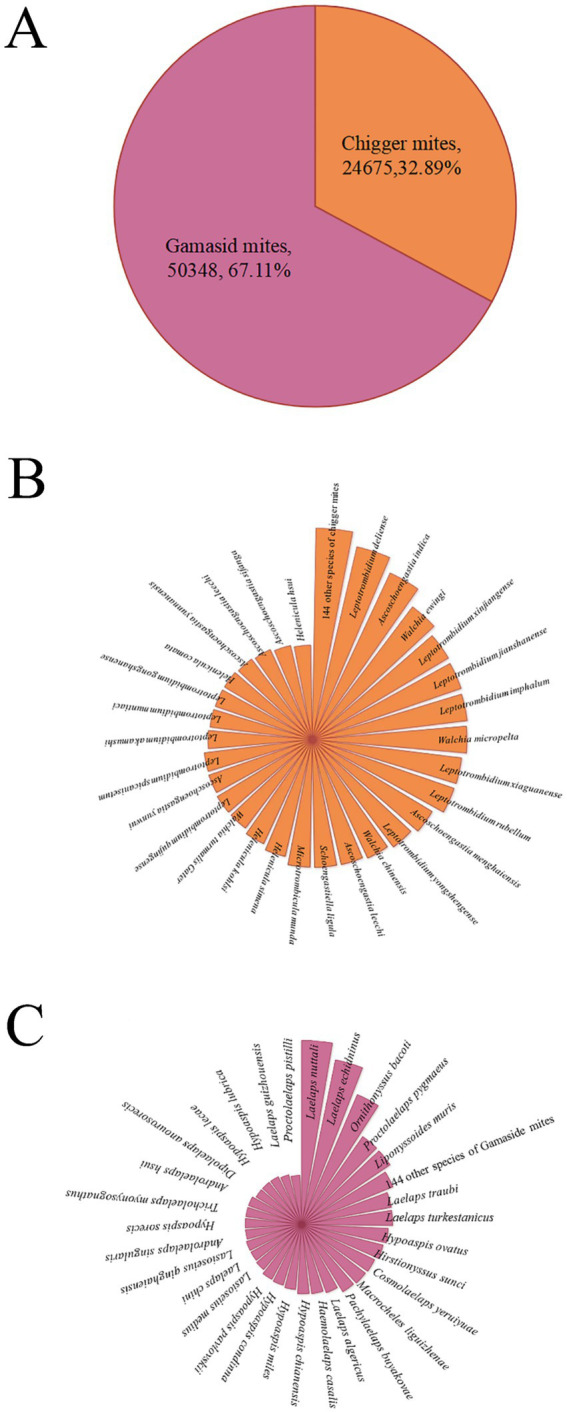
Composition of the mites on *R. tanezumi* in southwest China, 2001–2022 [annotations: **(A)**. Two groups of mites; **(B)**. species of the chigger mites; **(C)**. species of the gamasid mites. The radius length of each small sector in B and C represents the number of corresponding mite species].

A total of 75,023 mites were collected from 3,114 rat hosts (*R. tanezumi*) and identified, comprising 252 species, 46 genera, and 12 families. This included 173 species of chigger mites from 19 genera in two families and 79 species of gamasid mites from 27 genera in 10 families. Although there were more species of the chigger mites (173 species) than the gamasid mites (79 species), the individual constituent ratio (*C_r_*) of the gamasid mites (*C_r_* = 67.11%, 50,348/75023) was significantly higher than that of the chigger mites (*C_r_* = 32.89%, 24,675/75023). Among the 19 genera and two families of the chigger mites, the majority of the species (71) and individuals (14316) belonged to the genus *Leptotrombidium* in the family Trombiculidae. Among the 27 genera and 10 families of the gamasid mites, the majority of the species (12) and individuals (45454) belonged to the genus *Laelaps* in the family Laelapidae (Table 1; Figure 2). Among the 252 identified mite species, there were 22 vector species capable of serving as vectors or potential vectors for scrub typhus, HFRS, and other zoonotic diseases (zoonoses). These 22 vector mite species included 17 species of the chigger mites and five species of the gamasid mites. The 17 vector species of the chigger mites were as follows: *Leptotrombidium deliense* (Walch, 1922), *L. rubellum* (Wang and Liao, 1984), *L. akamushi* (Barumpt, 1910), *L. pallidum* (Nagayo et al., 1919), *L. scutellare* (Nagayo et al., 1921), *L. wenense* (Yang et al., 1959) (synonyms: *L. kaohuense*, *L. kaohuensis* or *L. gaohuensis*), *L. sialkotense* (Vercammen-Grandjean and Langston) (synonym: *L. jishoum*), *L. yui* (Chen and Hsu, 1955), *Ascoschoengastia indica* (Hirst, 1915), *Walchia chinensis* (Chen and Hsu, 1955), *Odontaearus majestivus* (Chen and Hsu, 1955), *L. guzhangense* (Wang et al., 1985), *L. apodeme* (Wen and Sun, 1984), *L. intermedium* (Nagayo et al., 1920), *L. fuji* (Kuwata et al., 1950), *L. imphalum* (Vercammen-Grandjean and Langston, 1975), and *W. pacifica* (Chen and Hsu, 1955). These species are known to act as vectors or potential vectors for scrub typhus. The five vector species of the gamasid mites were as follows: *Ornithonyssus bacoti* (Hirst, 1913), *Haemolaelaps glasgowi* (Ewing 1925), *Tricholaelaps myonysognathus* (Grochovskaya and Nguen-Xuan-Hoe, 1961), *Eulaelaps stabularis* (Koch, 1836), and *H. casalis* (Berlese, 1887). These can serve as vectors or potential vectors for rickettsialpox, HFRS, and other zoonoses (10, 11, 33, 35, 46, 47).

**Table 1:** Results of the taxonomic identification of the mites on *R. tanezumi* in southwest China (2001–2022).

Two groups of mites	Family and genus names of mites	No. of mite individuals	No. of mite species
Chigger mites	Family Trombiculidae (Ewing, 1944)	24,616	167
	Genus *Leptotrombidium* (Nagayo et al., 1916)	14,316	71
	Genus *Trombiculindus* (Radford, 1948)	21	6
	Genus *Neotrombicula* (Hirst, 1925)	3	3
	Genus *Chiroptella* (Vercammen-Grandjean, 1960)	6	1
	Genus *Lorillatum* (Nadchatram, 1963)	22	2
	Genus *Microtrombicula* (Ewing, 1950)	310	2
	Genus *Eutrombicula* (Ewing, 1938)	6	2
	Genus *Helenicula* (Audy, 1954)	673	14
	Genus *Doloisia* (Oudemans, 1910)	21	5
	Genus *Cheladonta* (Lipovsky et al., 1955)	6	1
	Genus *Ascoschoengastia* (Ewing, 1945)	5,216	12
	Genus *Walchiella* (Fuller, 1952)	46	2
	Genus *Herpetacarus* (Vercammen-Grandjean, 1960)	12	7
	Genus *Euschoengastia* (Ewing, 1938)	1	1
	Genus *Walchia* (Ewing, 1931)	3,499	19
	Genus *Schoengastiella* (Hirst, 1915)	318	3
	Genus *Gahrliepia* (Oudemans, 1912)	105	16
	Family *Leeuwenhoekiidae* (Womersley, 1944)	35	6
	Genus *Odontacarus* (Ewing, 1929)	17	4
	Genus *Chatia* (Brennan, 1946)	18	2
Gamasid mites	Family *Laelapidae* (Berlese, 1892)	46,642	42
	Genus *Laelaps* (Koch, 1836)	45,454	12
	Genus *Hypoaspis* (Canestrini, 1885)	384	12
	Genus *Proctolaelaps* (Berlese, 1923)	434	3
	Genus *Cosmolaelaps* (Berlese, 1892)	100	2
	Genus *Pachylaelaps* (Berlese, 1888)	92	2
	Genus *Haemolaelaps* (Berlese, 1910)	58	3
	Genus *Androlaelaps* (Berlese, 1903)	48	2
	Genus *Dipolaelaps* (Zemskaya and Pionkovskaya, 1960)	20	1
	Genus *Gymnolaelaps* (Berlese, 1903)	10	1
	Genus *Tricholaelaps* (Vitzthum, 1926)	24	1
	Genus *Qinghailaelaps* (Gu and Yang, 1984)	14	1
	Genus *Mysolaelaps* (Fonseca, 1935)	2	1
	Genus *Ololaelaps* (Berlese, 1904)	2	1
	Family *Blattisocidae* (Garman, 1948)	92	10
	Genus *Lasioseius* (Berlese, 1916)	92	10
	Family *Haemogamasidae* (Oudemans, 1913)	32	6
	Genus *Haemogamasus* (Berlese 1889)	32	6
	*Eulaelaps* (Berlese, 1903)	28	4
	Family *Macrochelidae* (Vitzthum, 1930)	118	7
	Genus *Macrocheles* (Latreille, 1829)	116	6
	Genus *Glyptholaspis* (Filipponi and Pegazzano, 1960)	2	1
	Family *Dermanyssidae* (Kolenati, 1859)	3,416	5
	Genus *Hirstionyssus* (Fonseca 1948)	118	2
	Genus *Ornithonyssus* (Sambon, 1928)	2,886	1
	Genus *Liponyssoides* (Hirst, 1913)	408	1
	Genus *Allodermanyssus* (Ewing, 1923)	4	1
	Family *Parasitidae* (Oudemans, 1901)	12	1
	Genus *Vulgarogamasus* (Tichomirov, 1969)	12	1
	Family *Ascidae* (Voigts and Oudemans, 1905)	2	1
	Genus *Asca* (Heyden, 1826)	2	1
	Family *Rhodacaridae* (Oudemans, 1902)	2	1
	Genus *Rhodacarellus* (Willmann, 1936)	2	1
	Family *Eviphididae* (Berlese, 1913)	2	1
	Genus *Eviphis* (Berlese, 1903)	2	1
	Family *Phytoseiidae* (Berlese, 1913)	2	1
	Genus *Amblyseius* (1915)	2	1
	Total	75,023	252

### 3.3 Infestation and community indexes of the mites on *Rattus tanezumi*

More than half of the *R. tanezumi* rats were infested with the mites, showing a high overall prevalence (*P_m_* = 71.77%), overall mean abundance (*MA* = 24.09 mites/per examined rat), and overall mean intensity (*MI* = 33.57 mites/per infested rat). The infestation indexes (*P_m_*, *MA*, and *MI*) for the gamasid mites were higher than those for the chigger mites (*p* < 0.05). However, the Shannon–Wiener diversity index (*H′*), Pielou’s evenness (*E*), and species richness index (*M_f_*) for the chigger mite community were higher than those for the gamasid mite community (Table 2).

**Table 2:** Infestation and community indexes of the mites on *R. tanezumi* in southwest China (2001–2022).

Mite groups	No. of mites	Infestation indexes of mites				Community indexes of mites			
		*C_r_*	*P_m_*	*MA*	*MI*	*H’*	*E*	*D*	*M_f_*
Chigger mites	24,675	32.89	29.96	7.92	26.45	2.82	0.55	0.13	21.38
Gamasid mites	50,348	67.11	54.30	16.17	29.77	1.11	0.25	0.47	9.70
Total mites	75,023	100.00	71.77	24.09	33.57	2.30	0.42	0.23	31.20

### 3.4 Dominant mite species and their infestation indexes

Of the 252 mite species identified, 12 were dominant species, including nine species of the chigger mites and three species of the gamasid mites (Table 3). Among the nine dominant species of the chigger mites, *Leptotrombidium deliense* had the highest prevalence (*P_m_* = 13.49%) and mean abundance (*MA* = 2.34) (*p* < 0.05). Among the three dominant species of the gamasid mites, *Laelaps echidninus* had the highest prevalence (*P_m_* = 36.90%, *p* < 0.05) and *L. nuttalli* had the highest mean intensity (*MI* = 30.54, *p* < 0.05) (Table 3). The 12 dominant mite species included six vector species (four chigger species and two gamasid mite species), which are the primary, secondary, or potential vectors for scrub typhus, rickettsialpox, HFRS, and other zoonoses (Table 3) (10, 11, 33, 35, 46, 47).

**Table 3:** Infestation indexes of the 12 dominant species of the mites on *R. tanezumi* in southwest China (2001–2022).

Names of dominant mite species	No. of mites	Constituent ratios (*C_r_*) and infestation indexes (*P_m_*, *MA*, *MI*) of mites				
		*C_r_* (%) in a single group of mites	*C_r_* (%) in two groups of mites	*P_m_*, %	*MA*	*MI*
Chigger mites						
Leptotrombidium deliense**	7,282	29.51	9.71	13.49	2.34	17.76
Ascoschoengastia indica*	3,882	15.73	5.17	3.78	1.25	32.90
*Walchia ewingi*	1759	7.13	2.34	2.67	0.56	21.19
*Leptotrombidium xinjiangense*	1,241	5.03	1.65	6.33	0.40	6.30
*Leptotrombidium jianshanense*	1,169	4.74	1.55	5.40	0.38	6.53
Leptotrombidium imphalum*	1,098	4.45	1.46	0.45	0.35	78.43
*Walchia micropelta*	1,033	4.19	1.38	0.29	0.33	11.11
*Leptotrombidium xiaguanense*	855	3.47	1.14	7.29	0.03	3.77
Leptotrombidium rubellum**	836	3.39	1.10	3.79	0.27	7.08
Gamasid mites						
*Laelaps nuttali*	31,940	63.44	42.57	33.59	10.26	30.54
*Laelaps echidninus* *	13,052	25.92	13.40	36.90	4.19	11.36
*Ornithonyssus bacoti* **	2,886	5.73	3.85	5.30	0.93	17.49

Annotations: (1) ** represents the main vector mite species, and * represents the secondary or potential vector mite species (10, 11, 33, 35, 46, 47). (2) The “C_r_ (%) in a single group of mites” refers to the constituent ratio (C_r_) of each mite species within one group of mites (either chigger mites or gamasid mites). The “C_r_ (%) in two groups of mites” refers to the constituent ratio (C_r_) of each mite species across both groups of mites together (chigger mites + gamasid mites).

### 3.5 Predictive model for the risk of the chigger mite infestation

In the multiple logistic regression analysis, the explanatory variables (independent variables) included eight environmental factors and three host factors, and the variable assignment is listed in Table 4. The relative fatness that lacked statistical significance was removed based on the backward stepwise multiple logistic regression analysis. The remaining ten risk factors were included in the multiple logistic regression model. The Hosmer–Lemeshow goodness-of-fit test showed a good model fit (*p* = 0.12). The results showed that temperature, relative humidity, landscape, longitude, latitude, habitat, and host age were the risk factors for the chigger mite infestation (Table 5). Based on the above analyses, risk scores (*S_c_*) for the seven risk factors were calculated using a scoring assessment system (Table 6). A predictive model for the chigger mite infestation on *R. tanezumi* was established as follows: ${P_{1}}^{\prime }=\frac{1}{1+\exp^{\left[-\left(-3.61+0.41S_{c1}\right)\right]}}$ . In the predictive model, *P’_1_* represents the risk probability of each rat host (*R. tanezumi*) being infested with chigger mites.

**Table 4:** Variable assignment in the multiple logistic regression analysis of the chigger mite infestation on *R. tanezumi* in southwest China (2001–2022).

Independent variables	Variable assignment
Temperature	0 = Temperature < =10°C; 1 = 10°C < Temperature < =20°C; 2= > 20°C
Precipitation	0 = Precipitation<90 mm; 1 = Precipitation> = 90 mm
Relative humidity	0 = Relative humidity<70%; 1 = Relative humidity> = 70%
Landscape	0 = Flatland; 1 = Mountainous
Altitude	0 Altitude>1,000 m; 1 Altitude<=1,000 m
Longitude	0 Longitude<100° E; 1 Longitude> = 100° E
Latitude	0 = Latitude>27° N; 1 Latitude<=27° N
Habitat	0 = Indoor; 1 = Outdoor
Relative fatness	Continuous variable
Sex	0 = Female; 1 = Male
Age	0 = Juvenile; 1 = Adult

**Table 5:** Results of the multiple logistic regression analysis of the chigger mite infestation on *R. tanezumi* in southwest China (2001–2022).

Variable	*β*	*SE*	Wald	df	Sig.	*OR*	95% *CI*	
							Lower	Upper
Temperature								
<=10°C						1.00		
10 < Temperature < =20°C	0.49	0.18	7.81	1.00	<0.01	1.63	1.16	2.30
>20°C	1.03	0.20	26.15	1.00	<0.01	2.81	1.89	4.17
Precipitation								
<90 mm						1.00		
> = 90 mm	0.14	0.19	0.59	1.00	0.44	1.16	0.80	1.67
Relative humidity								
<70%						1.00		
> = 70%	1.02	0.16	39.19	1.00	<0.01	2.78	2.02	3.82
Landscape								
Flatland						1.00		
Mountainous	0.79	0.17	21.78	1.00	<0.01	2.20	1.58	3.06
Altitude								
>1,000 m						1.00		
<=1,000 m	0.17	0.17	1.02	1.00	0.31	1.19	0.85	1.67
Longitude								
<100° E						1.00		
> = 100° E	0.41	0.41	8.58	1.00	<0.01	1.52	1.15	2.00
Latitude								
>27° N						1.00		
<=27° N	0.52	0.22	5.66	1.00	0.02	1.68	1.10	2.59
Habitat								
Indoor						1.00		
Outdoor	0.97	0.13	56.48	1.00	<0.01	2.63	2.04	3.39
Sex								
Female						1.00		
Male	0.27	0.15	3.36	1.00	0.07	1.31	0.98	1.74
Age								
Juvenile						1.00		
Adult	0.79	0.16	24.32	1.00	<0.01	2.21	1.66	3.02

**Table 6:** Risk scores (*S_c_*) for the chigger mite infestation on *R. tanezumi* in southwest China (2001–2022).

Variables	*β*	Scores (*S_n_*)
Temperature (10°C < Temperature < =20°C)	0.49	1
Temperature (>20°C)	1.03	3
Relative humidity (> = 70%)	1.02	2
Landscape (Mountainous)	0.79	2
Longitude (> = 100° E)	0.41	1
Latitude (<=27° N)	0.52	1
Habitat (outdoor)	0.97	2
Age (adult)	0.79	2

### 3.6 Predictive model for the risk of the gamasid mite infestation

The variable assignment is listed in Table 7. Six factors that lacked statistical significance were removed, and five risk factors were included in the multiple logistic regression model. The five risk factors were temperature, relative humidity, altitude, habitat, and host sex. The Hosmer–Lemeshow test indicated a good fit (*p* = 0.60) for the established multiple logistic regression model. All *OR* values for the different factors are shown in Table 8. The results showed that temperature, relative humidity, altitude, habitat, and host sex were the risk factors for the gamasid mite infestation. Based on the above analyses, risk scores (*S_c_*) for the five risk factors were calculated using a scoring assessment system (Table 9), and a predictive model for the gamasid mite infestation on *R. tanezumi* was developed as follows: ${P_{2}}^{\prime }=\frac{1}{1+\exp^{\left[-\left(-1.13+0.35S_{c2}\right)\right]}}$ . In the predictive model, *P’_2_* represents the risk probability of each rat host (*R. tanezumi*) being infested with gamasid mites.

**Table 7:** Variable assignment in the multiple logistic regression analysis of the gamasid mite infestation on *R. tanezumi* in southwest China (2001–2022).

Independent variables	Variable assignment
Temperature	0 = Temperature < 20°C; 1= > =20°C
Precipitation	0 = Precipitation<90 mm; 1 = Precipitation> = 90 mm
Relative humidity	0 = Relative humidity<70%; 1 = Relative humidity> = 70%
Landscape	0 = Flatland; 1 = Mountainous
Altitude	0 Altitude>1,000 m; 1 Altitude<=1,000 m
Longitude	0 Longitude>100° E; 1 Longitude<=100° E
Latitude	0 = Latitude>27° N; 1 Latitude<=27° N
Habitat	0 = Indoor; 1 = Outdoor
Relative fatness	Continuous variable
Sex	0 = Female; 1 = Male
Age	0 = Juvenile; 1 = Adult

**Table 8:** Results of the multiple logistic regression analysis of the gamasid mite infestation on *R. tanezumi* in southwest China (2001–2022).

Variable	*β*	*SE*	Wald	df	Sig.	*OR*	95% *CI*	
							Lower	Upper
Temperature								
<20°C						1.00		
> = 20°C	1.19	0.12	95.48	1.00	<0.01	3.30	2.60	4.19
Relative humidity								
<70%						1.00		
> = 70%	1.35	0.10	167.58	1.00	<0.01	3.86	3.15	4.74
Altitude								
>1,000 m						1.00		
<=1,000 m	0.51	0.10	27.94	1.00	<0.01	1.66	1.38	2.00
Habitat								
Indoor						1.00		
Outdoor	0.53	0.13	17.53	1.00	<0.01	1.69	1.32	2.17
Sex								
Female						1.00		
Male	0.35	0.13	7.76	1.00	0.01	1.42	1.11	1.83

**Table 9:** Risk scores (*S_c_*) for the gamasid mite infestation on *R. tanezumi* in southwest China (2001–2022).

Variables	*β*	Scores (*S_n_*)
Temperature (> = 20°C)	1.19	3
Relative humidity (> = 70%)	1.35	4
Altitude (<=1,000 m)	0.51	1
Habitat (outdoor)	0.53	2
Sex (male)	0.35	1

### 3.7 Predictive model for the co-infestation risk of both mite groups

The variable assignment is listed in Table 10. Three factors without statistical significance were removed, and eight risk factors were included in the multiple logistic regression model. The eight risk factors were temperature, precipitation, relative humidity, landscape, altitude, longitude, habitat, and host sex (Table 11). The Hosmer–Lemeshow test showed a good fit (*p* = 0.34) for the established multiple logistic regression model, and all *OR* values for the different factors are shown in Table 11. The results showed that temperature, precipitation, relative humidity, landscape, altitude, habitat, host sex, and host age were the risk factors for the co-infestation of the two mite groups. Based on the above analyses, risk scores (*S_c_*) for the eight risk factors were calculated using a scoring assessment system (Table 12), and a predictive model for the co-infestation of the two mite groups on *R. tanezumi* was established as follows: ${P_{3}}^{\prime }=\frac{1}{1+\exp^{\left[-\left(-1.96+0.37S_{c3}\right)\right]}}$ . In the predictive model, *P’_3_* represents the risk probability of each rat host (*R. tanezumi*) being infested with both mite groups.

**Table 10:** Variable assignment in the multiple logistic regression analysis of the combined infestation of the two mite groups on *R. tanezumi* in southwest China (2001–2022).

Independent variables	Variable assignment
Temperature	0 = Temperature < 20°C; 1= > =20°C
Precipitation	0 = Precipitation<90 mm; 1 = Precipitation> = 90 mm
Relative humidity	0 = Relative humidity<70%;1 = Relative humidity> = 70%
Landscape	0 = Flatland; 1 = Mountainous
Altitude	0 Altitude>1,000 m; 1 Altitude<=1,000 m
Longitude	0 Longitude>100° E; 1 Longitude<=100° E
Latitude	0 = Latitude>27° N; 1 Latitude<=27° N
Habitat	0 = Indoor; 1 = Outdoor
Relative fatness	Continuous variable
Sex	0 = Female; 1 = Male
Age	0 = Juvenile; 1 = Adult

**Table 11:** Results of the multiple logistic regression analysis of the combined infestation of the two mite groups on *R. tanezumi* in southwest China (2001–2022).

Variable	*β*	*SE*	Wald	df	Sig.	*OR*	95% *CI*	
							Lower	Upper
Temperature								
<20°C						1.00		
> = 20°C	1.46	0.12	155.56	1.00	<0.01	4.30	3.42	5.41
Precipitation								
<90 mm						1.00		
> = 90 mm	0.99	0.15	45.18	1.00	<0.01	2.70	2.02	3.61
Relative humidity								
<70%						1.00		
> = 70%	1.21	0.13	84.89	1.00	<0.01	3.34	2.58	4.31
Landscape								
Flatland						1.00		
Mountainous	0.51	0.12	19.85	1.00	<0.01	1.45	1.10	1.90
Altitude								
>1,000 m						1.00		
<=1,000 m	0.63	0.14	19.49	1.00	<0.01	1.88	1.42	2.49
Habitat								
Indoor						1.00		
Outdoor	0.50	0.16	9.61	1.00	<0.01	1.65	1.20	2.26
Sex								
Female						1.00		
Male	0.45	0.15	8.81	1.00	<0.01	1.57	1.17	2.12
Age								
Juvenile								
Adult	0.37	0.14	7.14	1.00	0.01	1.45	1.10	1.90

**Table 12:** Risk scores (*S_c_*) for the combined infestation of the two mite groups on *R. tanezumi* in southwest China (2001–2022).

Variables	*β*	Scores
Temperature (>20°C)	1.46	4
Precipitation (> = 90 mm)	0.99	3
Relative humidity (> = 70%)	1.21	3
Landscape (mountainous)	0.51	1
Altitude (<=1,000 m)	0.63	2
Habitat (outdoor)	0.50	1
Sex (male)	0.45	1
Age (Adult)	0.37	1

### 3.8 Interspecific relationship among some vector mite species on *Rattus tanezumi*

**Figure 3:**
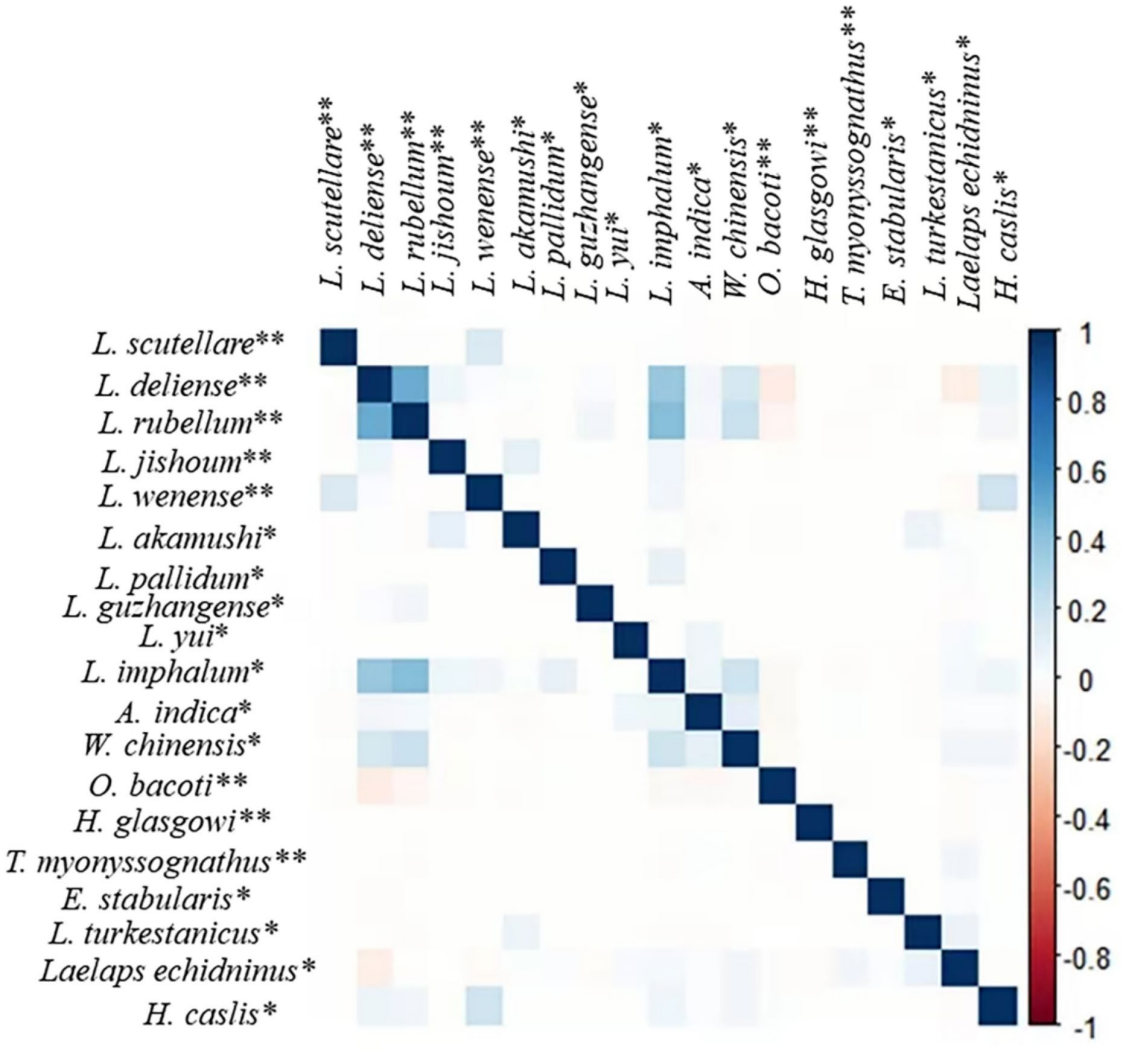
Interspecific relationships among some vector mite species on *R. tanezumi* in southwest China (2001–2022). Annotation: ** represents the main vector mite species, and * represents the secondary or potential vector mite species (10, 11, 33, 35, 46, 47).

The “corrplot” R package was used to analyze the interspecific relationship among 19 important vector mite species on *R. tanezumi*, with a confidence interval of 0.95. The result is shown in Figure 3. The 19 important mite species included 12 species of the chigger mites and seven species of the gamasid mites. The 12 species of the chigger mites were as follows: *L. scutellare*, *L. deliense*, *L. rubellum*, *L. jishoum*, *L. wenense*, *L. akamush*, *L. pallidum*, *L. guzhangense*, *L. yui*, *L. imphalum*, *A. indica*, and *W. chinensis*. The seven species of gamasid mites are *O. bacoti*, *H. glasgowi*, *T. myonyssognathus*, *E. stabularis*, *L. turkestanicus*, *L. echidninus*, and *H. caslis*. In Figure 3, the blue squares represent a positive correlation between any two mite species (values ranging from 0 to 1), while the pink squares represent a negative correlation (values ranging from 0 to −1). The color depth indicates the strength of the positive or negative correlation. The result showed that there was a positive correlation between any two of the following species: *L. deliense*, *L. rubellum*, and *L. imphalum* (Figure 3).

## 4 Discussion

As a very common rodent species, *R. tanezumi* is an important infectious source and reservoir host for many zoonotic diseases, such as scrub typhus and HFRS (17, 20). Southwest China is a significant focus for scrub typhus, HFRS, and other zoonotic diseases (15, 16). Rodent-associated mites (chigger mites and gamasid mites) found on *R. tanezumi* and other rodents can act as vectors or potential vectors for these zoonoses (48, 49). Therefore, studying the mites on *R. tanezumi* in southwest China holds considerable medical significance (50, 51). Although it is very common for chigger mites and gamasid mites to coexist on the body surface of the same rodent, the significant challenges in identifying species within these two mite groups (especially chigger mites) have often hindered their simultaneous study. As a result, previous reports have typically studied these two mite groups separately (20, 52). Based on the field surveys and taxonomic identification conducted in southwest China from 2001 to 2022, the present study investigated these two mite groups on the *R. tanezumi* rats as a whole for the first time. The results of the present study suggested that mite infestation is highly prevalent on *R. tanezumi* in southwest China, with a substantial mite burden (252 mite species and 75,023 mite individuals). The number of the chigger mite species (173) and gamasid mite species (79) found on *R. tanezumi—*a single rodent species—even exceeded the number of mite species found on several different host species across broader geographical regions of China. For example, 41, 53, and 81 species of chigger mites were recorded in Hubei Province, Fujian Province, and northwest China, which covers five provincial regions: Shanxi, Ningxia, Gansu, Qinghai, and Xinjiang (53–55). Similarly, 53, 21, and 36 species of gamasid mites were documented in Zhejiang, Chongqing, and northwest China (55–57). Southwest China encompasses a vast territory, complex and diverse landforms, rich vegetation and animal species, and a variety of climate types. These factors likely contribute to the high species diversity of mites found on *R. tanezumi* and other rodents in the region (51, 58). Different host species usually exhibit varying levels of susceptibility to mite and other ectoparasite infestations (9, 10). *Rattus tanezumi* is highly susceptible to mite infestation, which allows it to harbor a wide variety of mite species with a heavy infestation burden.

Of the 252 mite species identified, 22 species (17 chigger species and five gamasid mite species) can act as vectors or potential vectors for scrub typhus, HFRS, and other zoonotic diseases (10, 11, 33, 35, 46, 47). To date, six chigger species have been confirmed as the major vectors for scrub typhus in China, while several other chigger species are considered potential vectors. The six major vectors are *L. deliense*, *L. scutellare*, *L. rubellum*, *L. sialkotense*, *L. wenense*, and *L. insulare* (Wei et al., 1989). Evidence supporting their role as vectors comes from a series of epidemiological and experimental studies (10, 11, 33, 46). For example, large populations of these vector chigger mites have been identified in the foci of scrub typhus, and the seasonal fluctuation of the mites is highly consistent with the seasonal incidence of the disease (11, 27). *Orientia tsutsugamushi* (Ot), the causative agent of scrub typhus, has been repeatedly detected in vector chigger mites with natural infection. Ot can be successfully transmitted among laboratory rats and mice through the biting activity of the mites, which exhibit transovarial transmission characteristics (7, 9, 27, 33, 59). In addition, epidemiological and experimental evidence has also demonstrated that *L. scutellare* can effectively transmit *Hantaan virus* (hantavirus), the pathogen responsible for HFRS (27, 49). Similarly, through epidemiological and experimental studies, some gamasid mites have been proven to be vectors or potential vectors for rickettsialpox, HFRS, and other zoonotic diseases. For example, the pathogen of HFRS (hantavirus) has been detected or isolated from gamasid mites (12, 35, 47). Except for *L. insulare*, five of the six major vector chigger species in China were found on *R. tanezumi* in the present study. As a highly common rat species in human residential areas and nearby farmlands, *R. tanezumi* is closely associated with human life and agricultural activities (8, 20, 52). The presence of multiple vector mite species on *R. tanezumi* in southwest China increases the potential risk of transmitting scrub typhus, HFRS, and other zoonotic diseases from rats to humans through the biting activity of the mites. In addition, it increases the risk of focus persistence of the zoonoses in the region (11, 27, 60, 61).

Although multiple logistic regression analysis is widely used in epidemiology, economics, ecology, and other fields (39, 42, 62, 63), it has rarely been used in studies of rodent-associated mites (chigger mites and gamasid mites). The present study used the multiple logistic regression model to analyze the potential risk factors for mite infestation on *R. tanezumi* in southwest China for the first time. Some previous reports have shown that the infestation of ectoparasites can be simultaneously influenced by both environmental factors and host factors. The influencing factors and their effects on ectoparasites varied across different groups and species of ectoparasites (25, 64, 65). The results of the present study also suggested that a series of environmental factors and host factors can influence the infestation of mites on *R. tanezumi*. Although the effects of the risk factors on the infestation varied between the single mite group (chigger mites or gamasid mites) and the co-infestation of both mite groups together, temperature and relative humidity were the most important risk factors, with the highest risk scores *(S_c_)* (Tables 6, 9, 12). This is consistent with some previous reports on other ectoparasites (25, 66, 67). This result implies that in areas with higher temperatures and higher relative humidity, *R. tanezumi* is more likely to be infested with mites, which may further increase the potential risk of zoonosis transmission and focus persistence (25, 66). Based on multiple logistic regression, the present study established three predictive models for the infestation risk of chigger mites, gamasid mites, and both mite groups together on *R. tanezumi* in southwest China for the first time. In clinical practice, similar predictive models can be developed to estimate the risk probability of individual patients being infested with a specific disease (40, 42). The three predictive models established in the present study aimed to predict the risk probability of each *R. tanezumi* rat being infested with chigger mites, gamasid mites, or both mite groups together. The method and procedure used to establish these predictive models can also be applied to develop corresponding predictive models for other parasitic infections or infestations, including those caused by other ectoparasites (e.g., fleas and sucking lice) in vector surveillance programs.

The analysis using the “corrplot” R package revealed positive or negative correlations between any two of the 19 important vector mite species on *R. tanezumi* (Figure 3). A positive correlation existed between any two of the following species: *L. deliense*, *L. rubellum,* and *L. imphalum*. A positive correlation indicated that two mite species tend to co-exist on the same host, *R. tanezumi,* and that their host selection is the same or similar. A negative correlation indicated that two mite species have different preferences in the selection of hosts and that they may experience interspecifc competition or mutual repulsion in selecting hosts. *Leptotrombidium deliense*, *L. rubellum,* and *L. imphalum* are three important vectors for scrub typhus (10, 11, 59). The positive correlation between any two of these three vector species (Figure 3) revealed their tendency to co-exist on the same rat, *R. tanezumi*. The co-existence of more than two vector species on the same rat may also increase the potential risk of transmitting scrub typhus among rats and even from rats to humans.

## 5 Conclusion

*Rattus tanezumi* is highly susceptible to mite infestation, hosting a wide variety of mite species and multiple vector mite species. The species diversity of the chigger mites was higher than that of the gamasid mites; however, the infestation indexes of the gamasid mites were higher than those of the chigger mites. The presence of multiple vector mite species on *R. tanezumi* increases the potential risk of transmission and focus persistence of related zoonotic diseases. A series of environmental factors and host factors can influence the infestation of mites on *R. tanezumi*, and temperature and relative humidity are the most important risk factors. The established predictive models aimed to estimate the risk probability of each *R. tanezumi* individual being infested with mites. The positive and negative correlation among some vector mite species revealed their similar and differing preferences for the rat host, respectively.

## Data Availability

The raw data supporting the conclusions of this article will be made available by the authors, without undue reservation.
